# Chemical Modification and Processing of Chitin for Sustainable Production of Biobased Electrolytes

**DOI:** 10.3390/polym12010207

**Published:** 2020-01-14

**Authors:** Meriem Latifi, Azizan Ahmad, Hamid Kaddami, Nur Hasyareeda Hassan, Reiner Dieden, Youssef Habibi

**Affiliations:** 1 Faculty of Science and Technology, School of Chemical Sciences and Food Technology, Universiti Kebangsaan Malaysia, UKM Bangi 43600, Selangor DarulEhsan, Malaysia; latifimeriem@gmail.com (M.L.); azizan@ukm.my (A.A.); syareeda@ukm.edu.my (N.H.H.); 2 Laboratory of Organometallic and Macromolecular Chemistry, Faculty of Sciences and Technologies, Cadi Ayyad University, Avenue AbdelkrimElkhattabi, B.P. 549, Marrakech 40000, Morocco; 3 Materials Research and Technology Department (MRT), Luxembourg Institute of Science and Technology (LIST), 5 Avenue des Hauts-Fourneaux, L-4362 Esch-sur-Alzette, Luxembourg; Reiner.Dieden@list.lu

**Keywords:** chitin, Carboxymethylation, deacetylation, solid polymer electrolyte, ionic liquid

## Abstract

In the present work we report on the development of a novel and sustainable electrolyte based on chitin. Chitin biopolymer was carboxymethylated in simple, mild, and green conditions in order to fine-tune the final properties of the electrolyte. To this end, chitin was modified for various reaction times, while the molar ratio of the reagents, e.g., sodium hydroxide and monochloroacetic acid, was maintained fixed. The resulting chitin derivatives were characterized using various techniques. Under optimized conditions, modified chitin derivatives exhibiting a distinct degree of carboxymethylation and acetylation were obtained. Structural features, morphology, and properties are discussed in relation to the chemical structure of the chitin derivatives. For electrolyte applications, the ionic conductivity increased by three magnitudes from 10^−9^ S·cm^−1^ for unmodified chitin to 10^−6^ S·cm^−1^ for modified chitin with the highest degree of acetylation. Interestingly, the chitin derivatives formed free-standing films with and without the addition of up to 60% of ionic liquid, the ionic conductivity of the obtained solid electrolyte system reaching the value of 10^−3^ S·cm^−1^.

## 1. Introduction

Polymeric electrolytes are a class of polymeric materials widely used as an ionic conductive component in electrochemical devices. They can be processed in the form of dry solid polymer electrolytes (SPE), gel polymer electrolytes (GPE), and composite polymer electrolytes (CPE) [1]. Solid polymer electrolytes (SPE) as ionic conductive materials were first introduced by Armand et al. in 1979 [2]. Among the major trends in the sustainable development of SPE is the use of environmentally friendly processes and materials from renewable feedstock.

Ionic liquids (ILs) are molten salt or organic liquid salts, which possess properties distinct from conventional salts (e.g., NaCl) such as their ability to function as a solvent at room temperature, or their strong intermolecular [ion-ion] interactions that do not exist for other salts melted at high temperatures. SPEs based on ILs have been developed for energy storage and conversion materials and devices [3]. Based on past studies, the ionic conductivity of electrolytes containing ILs was in the range of 10^−2^−10^−5^ S·cm^−1^ [4,5,6,7,8]. It is worth highlighting that one of the most interesting properties of SPE-ILs is their electrochemical stability.

On the other hand, electrolytes based on polysaccharides, such as cellulose, agarose, chitosan, chitin, etc., are more and more used as a component of SPE as attested by recent studies [9]. These polysaccharides are long polymeric chains composed of carbohydrate molecules bound together by glycosidic linkages, making available along the polymer chain electron donating groups such as hydroxyl, amine, and carboxyl [10]. Moreover, polysaccharides, as semi-crystalline polymers, are able to offer solid electrolytes with good mechanical properties and high ion mobility thanks to their crystalline and amorphous phases, respectively.

Many studies have focused on the use of polysaccharides as fillers and/or matrices with different natural and synthetic polymers to investigate and develop new SPEs and CPEs (Composite Polymer Electrolytes). Azizi Samir et al. worked on composite polymer electrolytes based on poly(oxyethylene) reinforced with cellulose whiskers and they reported achieving a conductivity of about 3 × 10^−4^ S·cm^−1^ at 90 °C [11,12]. Subsequently, Yamazaki et al. developed a new cellulose-chitin blend based acidic gel electrolyte in ILs exhibiting improved conductive properties (0.578 S·cm^−1^ at 25 °C) [13]. In 2010, an electrolytic nanocomposite polymer composed of poly(ethylene oxide) and nanochitin for different concentrations of LiN(C_2_F_5_SO_2_)_2_ (LiBETI: bis(perfluoroethansulfonyl)imide) was prepared, the results of which demonstrated that the incorporation of nanochitin fillers improved the ionic conductivity by 10 fold [4].

Chitin (poly(b-(1-4)-*N*-acetyl-d-glucosamine) is the second affluent form of polysaccharide just after cellulose that is obtained from shells of marine organisms, e.g., shells of crustaceans, shrimps, and crabs [14]. It is worth noting that chitin possesses several interesting proprieties, e.g., physical, chemical, and biological properties, as reported in previous reviews [15,16,17]. The structure of chitin is very similar to cellulose and comprises *N*-Acetyl-d-glucosamine units which are associated with β-type (1–4) links [18]. Besides, this biopolymer has specific characteristics, such as its high molar mass [19] and low degree of deacetylation [20], as well as various crystalline states [21], that distinguish it from other natural polymers. These confer to chitin a number of specific physico-chemical properties such as accessibility of the internal sites in the macromolecular chains, the swelling properties in water, or diffusional properties, whereas biological properties consist of nontoxic, biodegradable, biocompatible, bioadhesion, and bioactivity [22]. Thus, chitin is widely applied in various medical treatments [23,24]. In CPE, nonwoven mats type separators based on regenerated chitin fibers showed excellent electrolyte-uptaking capability and Li-dendrite-mitigating performances in lithium batteries [25]. In SPE, a derivative of chitin has been evaluated; derivatization was mainly performed in order to improve the ionic conductivity and thermal stability [26].

In the present work, we modified chitin through carboxymethylation reaction in order to enhance its conductivity and also ensure better dispersion and/or solubility in water and polar solvent without too much compromising of its structural features and morphological aspects. Numerous studies have been carried out for the modification of polysaccharides via the classical carboxymethylation protocol to access, for example, carboxymethylcellulose [27,28,29], carboxymethyl starch [30] and carboxymethyl chitosan [31]. However, the production of carboxymethyl chitin is not straightforward compared to other polysaccharides modifications, because of the crystalline structure of chitin and also the high degree of acetylation. The application of classical experimental protocols usually used for the carboxymethylation of polysaccharides to chitin leads to the conversion of chitin, first to chitosan and then to carboxymethylated chitosan. Many serious studies in the literature have succeeded to prepare carboxymethylated chitin, but the protocols used remain tidy. The first studies on the carboxymethylation of chitin followed a protocol in which the reaction was performed in 40% sodium hydroxide solution at −20 °C for 12 h [32], then in recent studies, the conditions were optimized and less concentrated NaOH solutions (e.g., 8 wt.% NaOH in 4 wt.% urea) were used at −20 °C for 36 has a first step and then 15 °C for another 72 h [33]. Even under these conditions, the reaction remains complicated, as the risk of converting the chitin to chitosan is very high and the reaction time is very long. Therefore, one of the aims of the present study was to optimize a simple protocol and precisely to fine-tune the reaction time, the degree of substitution, and hence minimize the conversion of chitin into chitosan. The new chitin derivatives were then tested as SPE either alone or blended, for the first time to our knowledge, with ILs.

## 2. Materials and Methods

### 2.1. Materials

Chitin flakes were kindly supplied by chito-chemSdn Bhd., Port Klang, Malaysia (reference sample: CN-2014-A100). Ethanol, 95% of purity, was provided by SYSTERM ChemAR (Selangor, Malaysia). Sodium hydroxide, isopropanol, monochloroacetic acid, ionic liquid (1-butyl-3-methylimidazolium acetate), and acetic acid were purchased from Sigma-Aldrich (St. Louis, MO, USA). All materials were used without further purification.

### 2.2. Preparation of Carboxymethylatedchitin

10 g of chitin flakes were dispersed in aqueous 2-propanol 10% (*w*/*v*) and stirred at ambient temperature for 30 min to ensure good dispersion. 100 mL of NaOH 20% (*w*/*v*) aqueous solution was then added into the dispersion and the mixture was kept under stirring at room temperature for different times 15 min, 30 min and 45 min. Then 13.6 mL of 7.9 M solution of monochloroacetic acid in 2-propanol was slowly added to the mixture to reach a stoichiometric ratio (monochloroacetic acid)/(*N*-Acetyl-d-glucosamine units) of 2. After stirring at 35 °C for 4 h, the reaction was stopped by pouring the mixture into 3 times its volume of ethanol. The precipitated polymer was then filtered and rinsed with pure ethanol and finally dried in a desiccator under vacuum for 48 h. In the following, the unmodified and carboxymethylated chitin derivatives obtained are coined chitin, CMChit_15, CMChit_30, and CMChit_45, respectively (referring to the different reaction times of the pretreatment step with NaOH solution).

### 2.3. Films Processing

1 g of unmodified and carboxymethylated chitin derivatives were dissolved in 10 mL of 1% (*v*/*v*) aqueous solution of acetic acid. The solutions were cast in Petri dishes and left to dry at room temperature for 1 week to form self-standing films.

For the films obtained with ionic liquid, only the CMChit_30 sample was used to study the effect of the ionic liquid. Three films with different fractions of ionic liquid (1-butyl-3-methylimidazolium acetate ([Bmim][Ac])) were used: 20, 40 and 60 wt.% of the mass of CMChit_30 used. For each of these samples, CMChit_30 was dissolved in a 1% aqueous solution of acetic acid. After dissolution, the IL was added to the solution and left under stirring for 48 h. The solution was then cast in Petri dishes and left to dry for 1 week. All obtained films had thicknesses of 150 ± 30 µm.

### 2.4. Fourier Transformed Infra-Red (FTIR)

FTIR measurements were performed using a Perkin-Elmer 2000 spectrometer (Wellesley, MA, USA) with a spectral width ranging from 500 to 4000 cm^−1^, a resolution of 4 cm^−1^ and an accumulation of 32 scans. Degree of Acetylation was calculated by comparing the intensities of the FTIR bands at 1320 cm^−1^ and 1420 cm^−1^ following the method used in a previous report [34].

### 2.5. Nuclear Magnetic Resonance Spectroscopy (NMR)

Solid-state NMR spectra have been recorded on a Bruker Avance III HD 600 MHz spectrometer (Bruker, Rheinstetten, Germany) equipped with a 4 mm standard bore H-X probe using 4 mm zirconium oxide rotors with Kel-F caps at ambient temperature. ^13^C-DP MAS experiments were performed at a spinning speed of 14 kHz using a 90 degree pulse at 67.6 kHz and SPINAL64 dipolar decoupling [35] at 100 kHz and using a relaxation delay of 5 s, accumulating 4 k or 8 k transients. ^13^C-CP MAS spectra were recorded applying CP for 2 ms at a nutation frequency of 67.6 kHz using a 50–100% amplitude ramp on ^1^H centered on the first side-band, adding 512 or 2048 scans with a recycle delay of 5 s. Chemical shifts were referenced using adamantane as an external reference for tetramethylsilane (TMS), setting the CH_2_ signal to 38.48 ppm [36,37]. Data-processing, including peak deconvolution, was performed using ssNake software (Radboud University, Nijmegen, The Netherlands) [38]. The degree of substitution (DS) (which provide the amount of carboxymethyl moieties grafted on chitin macromolecular backbone) and the degree of acetylation (DA) (which provide the amount of the remaining acetyl groups on native chitin) were calculated from the peak areas of the peaks attributed to of ^13^C DP-MAS NMR spectrum using the integral of the peak corresponding to C1 as a reference, as this peak is well defined and not affected by the chemical modification [39].

### 2.6. X-ray Diffraction (XRD)

XRD was performed using a model D5000 Siemens apparatus (Billerica, MA, USA) to determine the crystallinity of chitin samples before and after the chemical modification. Data were collected for the range from 2θ from 5° to 80° at rate 0.05°·s^−1^ and the crystallinity indexes were calculated using the software EVA200 provided with the machine.

### 2.7. Field Emission Scanning Electron Microscopy (FE-SEM)

The morphologies and composition of the chitin and its derivatives were examined using FE-SEM-EDX Supra 55VP model (Carl Zeiss, Germany) at 20.0 kV using different magnifications. Samples were sputter-coated with gold prior to FE-SEM observation.

### 2.8. Thermogravimetric Analyses (TGA)

Thermal properties assessed by thermogravimetric analyses (TGA) were performed using LABSYSEVO from SETARAM instrument (Caluire-et-Cuire, France). Few milligrams of each sample were heated from 25 up to 800 °C at 10 °C·min^−1^ under a nitrogen flow.

### 2.9. Potentiometric Titration

Potentiometric Titration was conducted using the PHSJ-3F pH Meter apparatus (Columbus, OH, USA), 300 mg of chitin or carboxymethylated chitin were dispersed in 30 mL of HCl 0.1 M for 30 min and then was titrated with an aqueous solution of NaOH 0.1 M. The degree of substitution (DS) and degree of acetylation (DA) values of all samples (chitin and its derivatives) were calculated from the results of potentiometric titration according to the methods used, respectively, in previous reports [40,41].

### 2.10. Ionic Conductivity Measurements

Ionic conductivity measurements, performed on pellets for unmodified chitin (not forming films) or on films for carboxymethylated chitin derivatives, were carried out using a Versa Stat Conductivity Meter from Schlumberger Instrument (Berwyn, PA, USA). The measurement of impedance was performed in a frequency range between 10^−3^ and 10^6^ Hz. Before the impedance analysis was performed, the sample was first punched using the Facia Kit-hole 16 mm, placed between two stainless steel electrodes, and the measurements were performed at room temperature. Through this analysis, the available bulk resistances of electrolytic polymers are determined. It was obtained through the formation of a circle fit from the equivalence circuit analysis by using the Zview analyzer software (Berwyn, PA, USA). The conductivity values, (σ) of electrolytes have been calculated from the Equation (1).

σ = *t*/*A* × *R* (1)

With: the thickness of the sample (cm); *A*: the sample area (cm^2^); *R*: the resistance (Ω).

## 3. Results and Discussion

### 3.1. Preparation and Characterization of Chitin Derivatives

**Figure 1:**
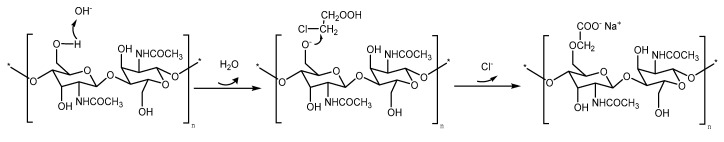
Reaction scheme for the carboxymethylation process on chitin.

The carboxymethylation of chitin requires a strong base such as sodium hydroxide to deprotonate its hydroxyl groups to form alkoxide anions. The carboxymethyl groups are then formed when reacted with monochloroacetic acid. The overall reaction of chitin is shown in the schema presented in Figure 1. The deacetylation of the acetylamide groups can occur to different extents depending on the conditions used for the carboxymethylation. It is noteworthy that a balanced control of the degree of carboxymethylation noted hereafter as the degree of substitution (DS) and the degree of acetylation (DA), is challenging when preparing carboxymethyl chitin, which we aim in the present work to control as much as possible.

**Figure 2:**
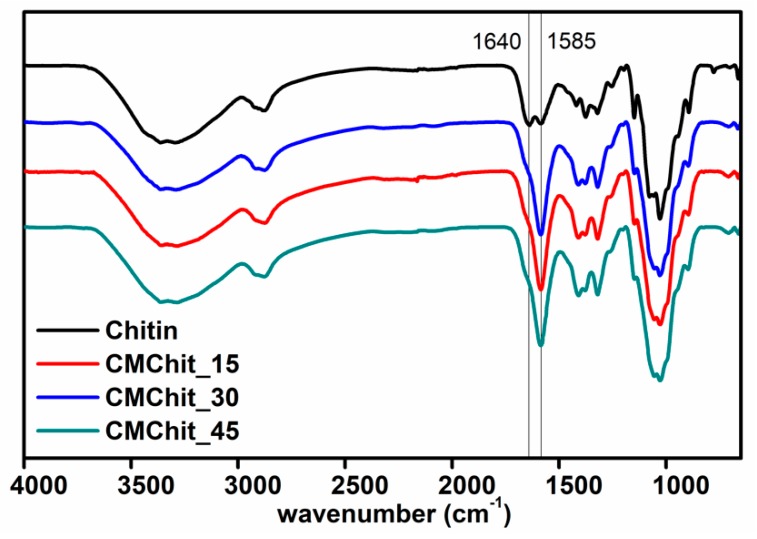
FTIR spectra of unmodified chitin and the different carboxymethylated derivatives obtained under different conditions.

Chitin samples were activated through deprotonation using alkaline pretreatment at various durations while carboxymethylation was performed at a fixed molar ratio of (monochloroacetic acid)/(*N*-Acetyl-d-glucosamine units) of 2. The success of the reaction was followed by FTIR. Figure 2 gathers the recorded FTIR spectra for unmodified chitin and the different derivatives, namely CMChit_15, CMChit_30, and CMChit_45. The wavenumber regions around 1640 and 1585 cm^−1^ are the characteristic bands of the amide I (C = O) and amide II (C-N and H-N) [42]. For the derivatives, the characteristic peak of sodium-carboxymethyl function overlaps with the characteristic band of amide II around 1585 cm^−1^, also explaining the strong increase of this band in all CMChit samples.

The presence of the 1640 cm^−1^ peak representing the amide I in the FTIR spectra in CMChit_15, CMChit_30, and CMChit_45 derivatives confirms the conservation of *N*-Acetyl-d-glucosamine units of the native chitin structure, although the degree of carboxymethylation is increasing, which demonstrates the appropriateness of the chosen reaction conditions to control the carboxymethylation of chitin while preserving the acetyl groups in the glucosamine units as much as possible [42].

**Figure 3:**
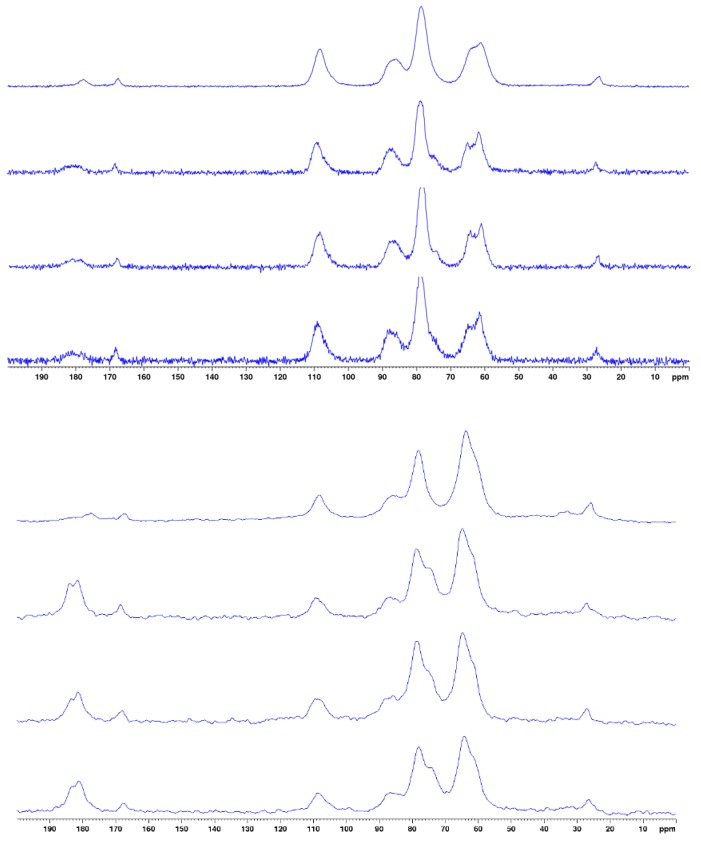
^13^C CP-MAS (**top**) and 13C DP-MAS (**bottom**) NMR spectra of unmodified chitin and carboxymethylated derivatives obtained under different conditions (from top to bottom: chitin, CMChit_15, CMChit_30 and CMChit_45).

The success of the carboxymethylation reaction was also checked by solid state ^13^C NMR spectroscopy and the recorded spectra are shown in Figure 3. These analyses were carried out to confirm the structural features and prospective changes of the chitin, also permitting us to determine the modification of the structural features of the chitin chains, namely the degree of substitution (DS) and the degree of acetylation (DA).

Table 1 lists the ^13^C NMR chemical shifts of the different carbons present in chitin and its derivatives according to [21]. As we can see in Figure 3, the spectrum of unmodified chitin shows, in addition to the characteristic peaks of chitin, a peak at 175 ppm, most probably resulting from slight oxidation occurring during the process of the extraction of chitin [43]. On the other hand, the peak at 26.31 ppm confirms the conservation of the acetyl group. On the other hand, the occurrence of the chemical modification (carboxymethylation) was clearly evidenced by the appearance of a new peak at 180 ppm in the spectra of the modified chitin, attributed to carboxylic carbons [43]. After carboxymethylation, the intensity of this peak increased together with the DS as determined by NMR (see Table 2).

The DS and DA were deduced from peak areas in DP-NMR spectra, obtained by deconvolution, of the peaks corresponding to the methylene groups of the carboxymethyl moieties and the acetyl groups of the N-glucosamine units, respectively. The integral of the peak attributed to the carbon C1 is used as a reference. These values were also determined by potentiometric measurements and FTIR analyses. All data are presented in Table 2 with all characteristics of these substrates.

**Figure 4:**
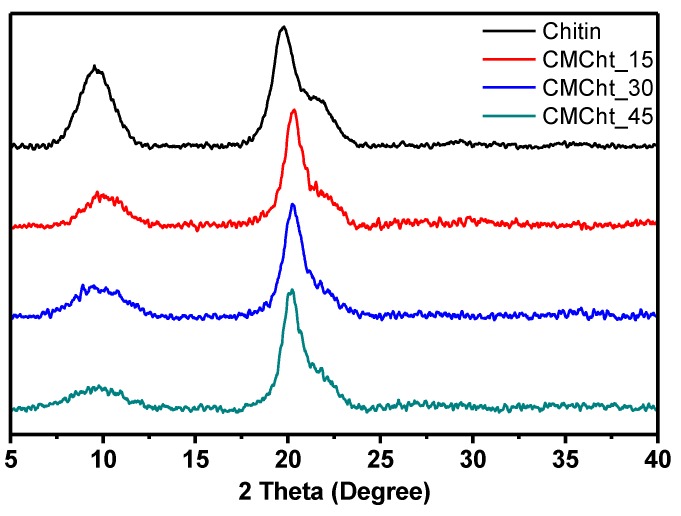
XRD Diffractograms of the unmodified chitin and different carboxymethylated derivatives obtained under different conditions.

The calculations of DA confirm that the chitin structure is predominant and derivatization from chitin to chitosan doesn’t occur under these reaction conditions. In fact, native chitin exhibits a DA around 82%, as determined by NMR. This DA decreases down to 70% for sample CMChit_30 while the modification is extended, achieving a higher amount of introduced carboxymethyl moieties of around 1.9 (DS). One can observe that the DA values measured by potentiometric titration and FTIR methods are slightly lower than those calculated from NMR analyses, yet the same trend is observed. For higher pretreatment times, CMChit presents high values of DS. This is certainly due to side chain relaxations which induce an overestimation of the integrations of the NMR peaks [44]. The XRD profile (Figure 4) of the unmodified chitin, carboxymethyl chitin derivatives obtained at different pretreatment times shows two diffraction peaks. The mean reflection peaks related to chitin are around 9.6° and 19.8°. The peak at 19.8° is characteristic of the strong hydrogen bond network present in the chitin structure [45]. The native chitin used in the present study exhibits a crystallinity index (CI) of 63%. After the chemical modification, the peak at 19.8° shifts to 20.1° and its intensity decreased, to different extents, in all derivatives depending on their DS. However, the peak at 9.6° becomes broader toward high values of 2θ and its greatly decreased intensity, which might be due to the deacetylation, might also suggest that a mild swelling of the crystallite in the native crystalline structure of the used chitin occurs during the carboxymethylation process [46]. A shoulder peak at 22.5° is observed in all samples, and its intensity decreases with the chemical modification. This peak is related to the presence of crystalline defects. The crystallinity indexes dropped from 63% to around 24–26% for all carboxymethylated derivatives, regardless of their DS, confirming the swelling of the native crystalline structure of the used chitin (see Table 2). All these observations corroborate previous results and confirm that the combination of the alkaline pretreatment and the carboxymethylation reaction induce the swelling of the crystallites in polysaccharides such as cellulose and chitin. This swelling phenomenon can evolve into crystal distortion or even destruction, and, in some cases, may induce polymorph conversion when harsh conditions are applied.

**Figure 5:**
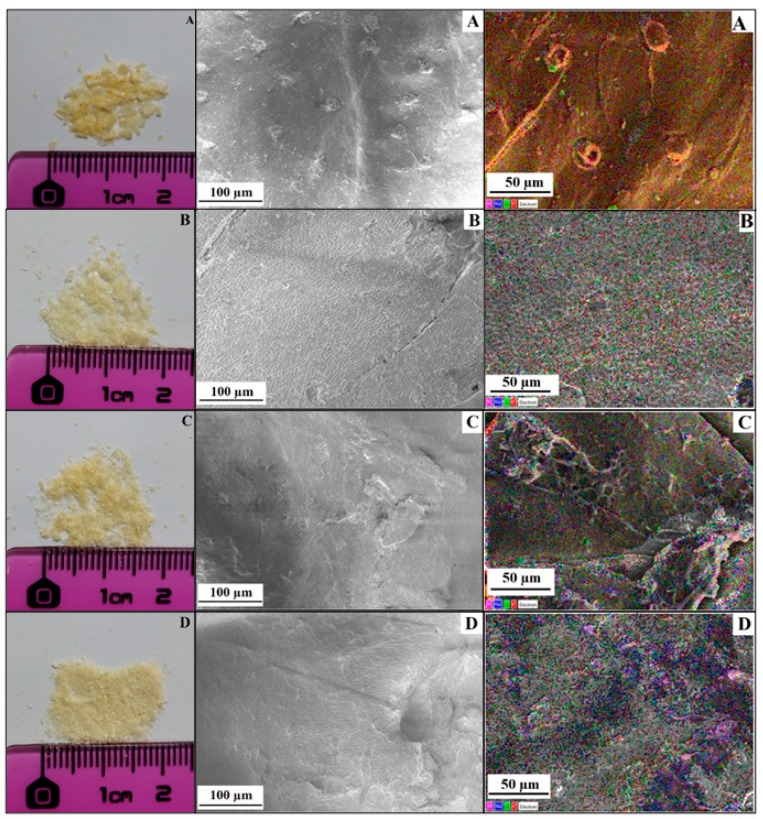
FE-SEM imaging (**middle**) and the corresponding EDX mapping (**right**) of unmodified chitin (**A**) and carboxymethylated derivatives (**B**): CMChit_15, (**C**): CMChit_30, (**D**): CMChit_45) obtained under different conditions. On the **left** are presented the optical photos of the corresponding flakes.

The visual aspect of the flakes of received chitin and chitin derivatives are shown in Figure 5 (to the left of the FE-SEM images) with a ruler scale. These pictures show a significant reduction of the size of flakes with the extent of the alkaline chemical pretreatment, which might suggest or confirm the significant change of the structure and properties of chitin. Observations have shown that the surface morphology of unmodified chitin flakes is smooth with the presence of structures or defects of about 25 µm in size, more or less homogeneously distributed at the surface of the flake. However, in chitin derivatives, the surface became waved and the emerging structures (or defects) shrank and subsequently disappeared for CMChit_30min and CMChit_45min, suggesting the diffusion of NaOH solution into the core of the flakes during the carboxymethylation reaction [42].

These ripples suggest an increase of the amorphousness of the biopolymer after the chemical treatments, as was confirmed by the XRD analyses of the crystalline index. On the right, in Figure 5, the EDX image of the unmodified chitin and chitin derivatives are presented (images A, B, C, and D). Unlike unmodified chitin (image A), the chitin derivatives (images B, C, and D) clearly show the presence of blue color, referring to the presence of sodium provided by carboxymethylated groups. On the other hand, in these images, one can observe an increase in the red and green colors, referring to the carbon and oxygen, respectively. All these observations, qualitatively, confirm the carboxymethylation of the chitin.

**Figure 6:**
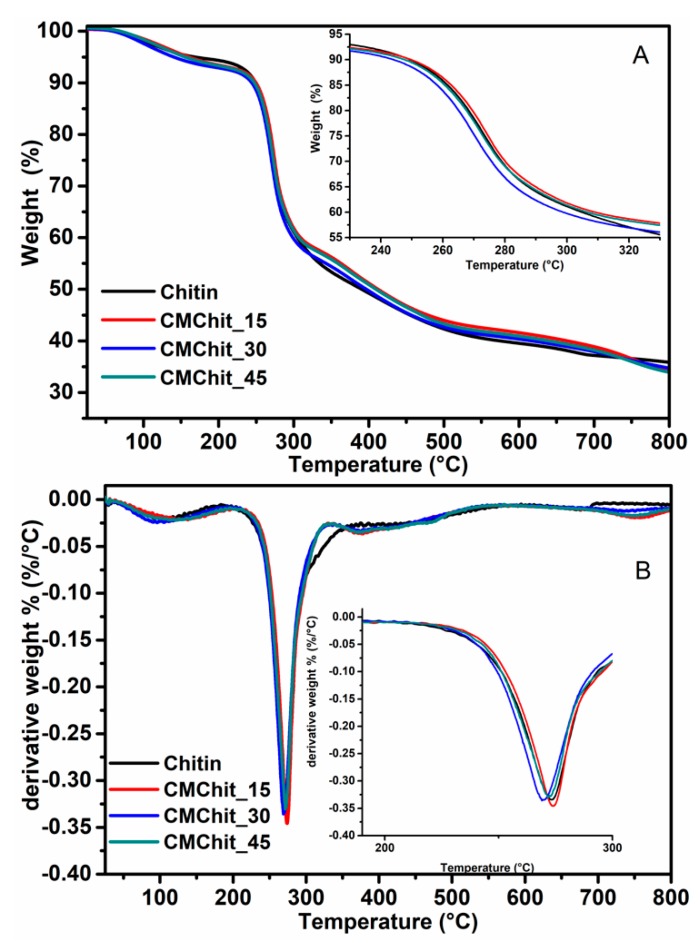
TGA thermograms (**A**) and corresponding first derivatives (**B**) of unmodified chitin and carboxymethylated derivatives obtained under different conditions.

The thermal behavior of native chitin, and after being modified at different degrees of substitution, was monitored by TGA, and the recorded curves of all samples are depicted in Figure 6. Two separate weight losses were detected in the curves of all samples. The first weight loss, occurring at the temperature range between 40 to 110 °C, is due to the evaporation of adsorbed residual water molecules that are well known to strongly attach to polysaccharides through hydrogen [47]. The second weight losses of the chitin and chitin derivatives, in the range of 200 to 215 °C, are due to the degradation of the polysaccharides. The degradation temperature (the onset point) seems to decrease slightly while comparing unmodified chitin and chitin derivatives (215 °C versus 210–200 °C) (Table 2). These observations suggest a decrease in the thermal stability of chitin derivatives compared to chitin. This decrease in thermal stability seems to be logical in view of the reduction of the crystalline index and the introduction of salt groups on the backbone of the chitin chain [48].

### 3.2. Films Processing and Characterization

**Figure 7:**
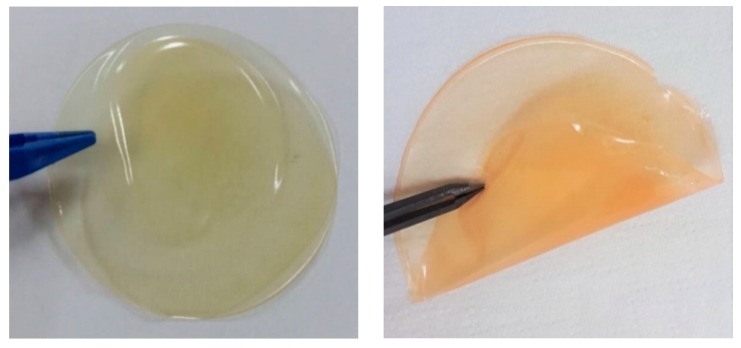
Optical photos of CMChit (**left**) and CMChit/IL films (**right**).

Before the processing of unmodified chitin and its carboxymethylated derivatives into films for further characterization as SPE, dispersion tests were performed in order to select a suitable system to process the films using solvent casting. Two solvent systems (neat water or 1% acetic acid aqueous solution) were tested for chitin and carboxymethylated chitin derivatives to get homogenous dispersion. The tests showed that the 1% acetic acid aqueous solution provides the best dispersion and then was used to process the samples into films by solvent casting. Figure 7 presents photos of the films of CMChit and CMhit/IL. Films with IL are more flexible compared to plain films with CMChit.

**Figure 8:**
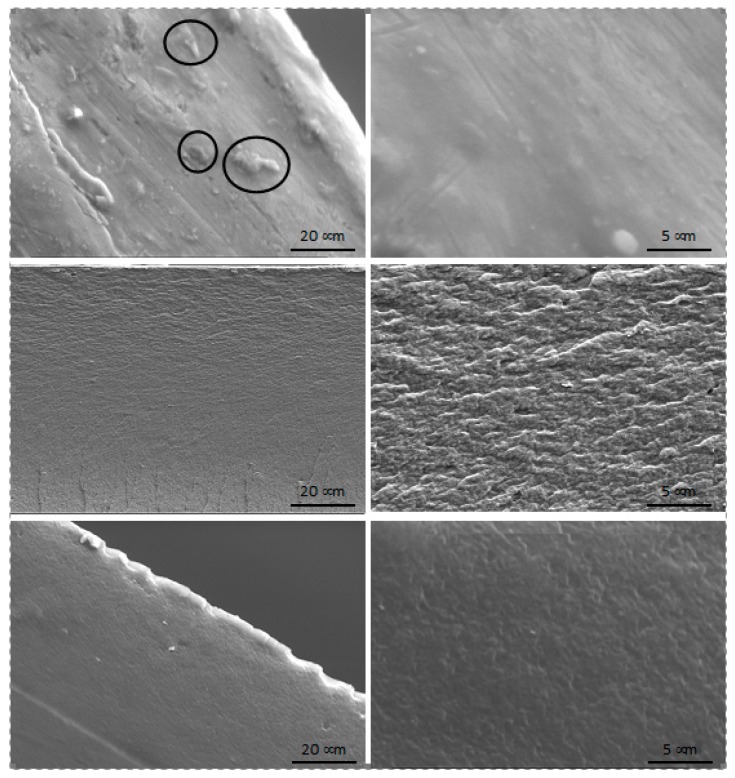
FE-SEM pictures of films based on carboxymethylated chitin derivatives (top to bottom CMChit_15, CMChit_30 and CMChit_45) obtained by solvent casting in 1% acetic acid aqueous solution.

Different carboxymethylated chitin samples obtained under different conditions of chemical modification (pretreatment step and duration of the reaction), namely CMChit_15, CMChit_30, and CMChit_45, were used to form the films. After solvent casting in 1% acetic acid aqueous solution, self-standing films were obtained and their morphologies were characterized by FE-SEM. Figure 8 shows the FE-SEM micrographs of the cross-section of resulting carboxymethylated chitin films with two different magnifications (1000× and 5000×). FE-SEM micrographs of CMChit_15 show small particles, surrounded with circles in Figure 6, corresponding to the insoluble parts of the flakes which remain in the casted film. However, for CMChit_30 and CMChit_45 the films exhibit smooth and homogeneous surfaces, confirming that these samples contain small crystallites surrounded by distorted chains that swell in the used solvent, therefore forming homogenous films.

The films prepared by the casting method were used to perform EIS analyses of different chitin derivatives, the recorded values of which are presented in Table 2. Note that because of the limited solubility of chitin, pellets were used instead of films, and the conductivity value is provided as indicative reference.

**Figure 9:**
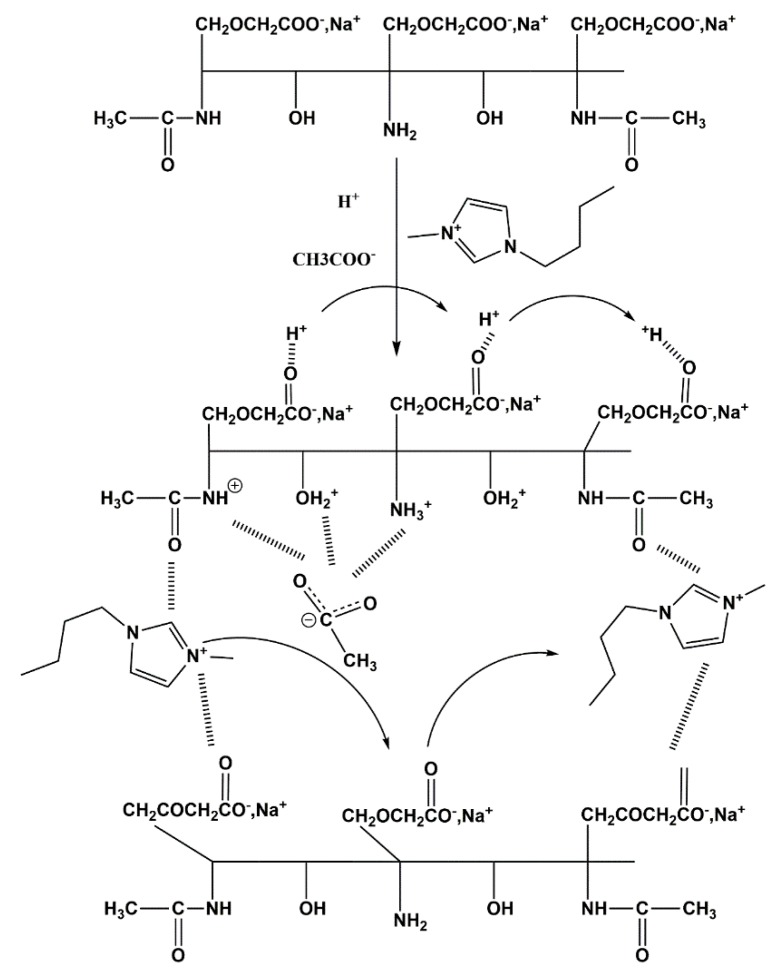
The proposed mechanisms of ion motion in (**top**) CMChit/Acetic Acid aqueous solution and in (**bottom**) CMChit/Bmim[Ac]/acetic acid system.

The ionic conductivity of the chitin derivatives increased by 3 orders of magnitude (i.e., in the logarithmic scale) compared to the starting material (chitin). The conductivity values achieved were 9.21 × 10^−6^ S·cm^−1^ for CMChit_15, 4.17 × 10^−6^ S·cm^−1^ for CMChit_ 30, and 3.08 × 10^−6^ S·cm^−1^ for CMChit_45 in comparison to 1.55 × 10^−9^ S·cm^−1^ recorded for chitin. The conductivity values achieved for carboxymethyl chitin derivatives were higher compared to pure chitin, which may be due to the ion movement through a hopping mechanism as proposed in scheme A of Figure 9. This ionic movement is enhanced in the carboxymethylated chitin thanks to the polarization of carboxymethyl groups due to the presence of the oxygen ion pairs. Moreover, chain flexibility and perhaps their entanglement induced by the decrease of the crystallinity may also favor the ion movement in the system [45]. The reduction of the conductivity from CMChit_15 to CMChit_45 seems as contradictory with the presence in the casted films of insoluble particles that should constrain the ionic conductivity. However, this evolution could be attributed to the increase of the degree of deacetylation and the increase of -NH2 group concentration. In fact, the -NH2 groups trap protons and slow down their movement [49]. This might also explain the fact that the values of ionic conductivity obtained are higher than those reported by Mobarak et al. for carboxymethyl chitosan prepared under the same conditions [45].

**Figure 10:**
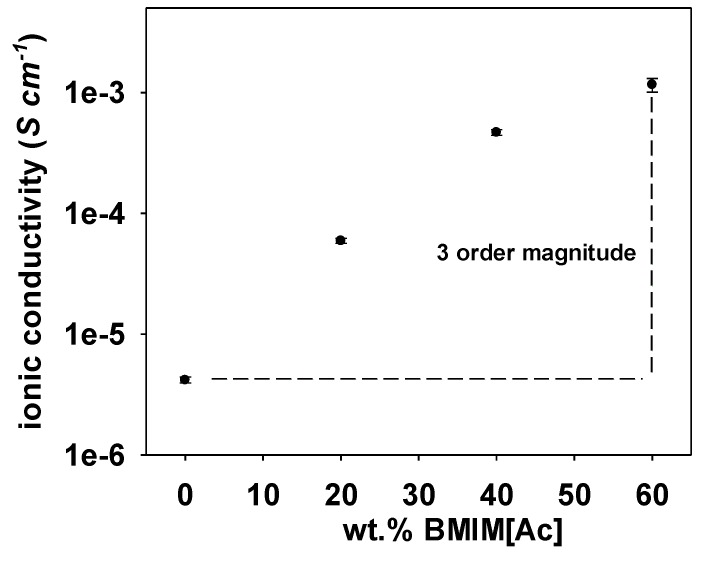
Ionic conductivity of carboxymethyl chitin CMChit_30 with different amounts of BMIM [Ac].

In order to enhance the ionic conductivity of these chitin-based electrolytes, different amounts ranging from 20% up to 60% of [Bmim][Ac] ionic liquid were added to the solution made of the sample with medium degree of modification, e.g., CMChit_30, in 1% acetic acid solution before casting into films. Surprisingly, even with high loadings of IL, the carboxymethylated chitin samples were able to form self-standing films indicating strong interactions between the used IL and chitin chains that are still under deep scrutiny by small angle X-ray diffraction and solid-state NMR. The ionic conductivity values measured for the different films are represented in Figure 10. These string interactions between ILs and chitin chains greatly improved the ionic conductivity of the membranes by increasing the number of mobile ions in the system as the suggested mechanism depicted in Figure 9 bottom. Indeed, the ionic conductivity increases with an increasing of the amount of [Bmim][Ac] and the effect of the ionic liquid can be detected starting from low loading, 20 wt.% [Bmim][Ac] as it increases the ionic conductivity by one order of magnitude (in logarithmic scale) to 5.92 × 10^−5^ S·cm^−1^. The highest ionic conductivity achieved, 1.16 × 10^−3^ S·cm^−1^ was attained at 60 wt.% of [Bmim][Ac] at room temperature. This value of ionic conductivity is higher compared to the value reported for chitosan loaded with the same amount of [Bmim][Ac], which was 5 × 10^−4^ S/cm [50]. Yet, an ionic conductivity value of the same order of magnitude was reported for fully carboxymethylated chitosan with the same load of ionic liquid (60 wt.% of [Bmim][Ac]) [51].

## 4. Conclusions

Results have convinced us that the green and optimized derivation method followed was successful to produce carboxymethyl chitin (CMChit). The FTIR, solid state ^13^C NMR, and potentiometric titrations proved the modification of chitin to CMChit with a preservation of the chitin amide functions depending on the extent of the alkaline pretreatment of the chitin, and this due to the presence of the bands 1640 cm^−1^ and 1585 cm^−1^ in FTIR, besides 27 ppm and 180 ppm in NMR. The XRD, FESEM-EDX and TGA analyses of the CMChit confirmed the modification by the changes occurred in the structure and the physico-chemical properties in comparison to the neat chitin. The ionic conductivity is increased after derivation and introduction of ionic liquid by more than 6 orders of magnitude to reach 1.16 × 10^−3^ S·cm^−1^ after the introduction of 60 wt.% of [Bmim][Ac]. These findings clearly show the high potential of these chitin derivatives (CMChit) as high performance, cheap, and environmentally friendly biopolymer electrolytes.

## Figures and Tables

**Table 1:** Observed ^13^C chemical shifts (ppm) in the CP/MAS NMR spectra for unmodified chitin and carboxymethylated derivatives obtained under different conditions. Attributions made according to Mi-Kyeong Jang et al. (Jang et al. 2004).

Chemical Shift (ppm)								
Samples	C1	C2	C3/C5	C4	C6	CH3	C = O *	C = O **
Chitin	105.2	57.8	75.46	83.5	59.9	24.0	164.8	174.8
CMChit_15	108.7	61.5	78.7	87.4	64.8	27.4	168.3	180.2
CMChit_30	108.6	61.5	78.7	87.4	64.8	27.2	168.3	180.7
CMChit_45	108.6	61.3	78.6	87.4	64.8	27.4	168.4	180.6

C = O * acetyl, C = O ** Carboxymethyl.

**Table 2:** Degree of substitution (DS) and degree of acetylation (DA) determined by potentiometric titration, FTIR, and NMR for chitin and chitin derivatives.

	Potentiometric Titration		FTIR Analyses	NMR		XRD	TGA		EIS
Samples	DS	DA	DA	DS	DA	CI (Crystalline Index)	Td (Onset) °C	T (Peak) °C	Ionic Conductivity S/cm
Chitin	-	69 ± 1	68	-	82	63%	215	273	1.55 × 10^−9^ *
CMChit_15	0.93 ± 0.05	64 ± 3	63	1.73	75	27%	215	273	9.21 × 10^−6^
CMChit_30	0.95 ± 0.05	60 ± 2	61	1.90	70	24%	215	270	4.17 × 10^−6^
CMChit_45	0.92 ± 0.10	51 ± 4	56	2.85	65	24%	210	272	3.08 × 10^−6^

* Test is performed on pellets of chitin flakes instead of films.

## References

[1] Stephan A.M., Nahm K.S. (2006). Review on composite polymer electrolytes for lithium batteries. Polymer.

[2] Armand M. (1983). Polymer solid electrolytes—An overview. Solid State Ion..

[3] Watanabe M., Thomas M.L., Zhang S., Ueno K., Yasuda T., Dokko K. (2017). Application of Ionic Liquids to Energy Storage and Conversion Materials and Devices. Chem. Rev..

[4] Angulakshmi N., Prem K.T., Sabu T., Manuel S.A. (2010). Ionic conductivity and interfacial properties of nanochitin-incorporated polyethylene oxide-LiN (C2F5SO2)2 polymer electrolytes. Electrochim. Acta.

[5] Leones R., Sentanin F., Rodrigues L.C., Marrucho I.M., Esperança J.M.S.S., Pawlicka A., Silva M.M. (2012). Investigation of polymer electrolytes based on agar and ionic liquids. Express Polym. Lett..

[6] Liew C.W., Ramesh S. (2013). Studies on ionic liquid-based corn starch biopolymer electrolytes coupling with high ionic transport number. Cellulose.

[7] Ou R., Xie Y., Shen X., Yuan F., Wang H., Wang Q. (2012). Solid biopolymer electrolytes based on all-cellulose composites prepared by partially dissolving cellulosic fibers in the ionic liquid 1-butyl-3- methylimidazolium chloride. J. Mater. Sci..

[8] Ramesh S., Shanti R., Morris E. (2013). Employment of [Amim] Cl in the effort to upgrade the properties of cellulose acetate based polymer electrolytes. Cellulose.

[9] Singh R., Reddy A., Bhattacharya B., Rhee H.-W., Varlikli C. (2016). Perspectives for solid biopolymer electrolytes in dye sensitized solar cell and battery application. Renew. Sustain. Energy Rev..

[10] Cǎpriţǎ R., Cǎpriţǎ A., Julean C. (2010). Biochemical Aspects of Non-Starch Polysaccharides. Sci. Paper. Anim. Sci. Biotechnol..

[11] Azizi Samir M.A.S., Alloin F., Dufresne A. (2005). Review of Recent Research Into Cellulose Whiskers, Their Properties and Their Application in Nanocomposite Field. Biomacromolecules.

[12] Azizi Samir M.A.S., Alloin F., Sanchez J.Y., Dufresne A. (2004). Cross-linked nanocomposite polymer electrolytes reinforced with cellulose whiskers. Macromolecules.

[13] Yamazaki S., Takegawa A., Kaneko Y., Kadokawa J.-I., Yamagata M. (2009). An acidic cellulose-chitin hybrid gel as novel electrolyte for an electric double layer capacitor. Electrochem. Commun..

[14] Li H., Greene L.H. (2010). Sequence and Structural Analysis of the Chitinase Insertion Domain Reveals Two Conserved Motifs Involved in Chitin-Binding. PLoS ONE.

[15] Rinaudo M. (2006). Chitin and chitosan: Properties and applications. Prog. Polym. Sci..

[16] Synowiecki J., Al-Khateeb N.A. (2003). Production, Properties, and Some New Applications of Chitin and Its Derivatives. Crit. Rev. Food Sci. Nutr..

[17] Zargar V., Asghari M., Dashti A. (2015). A Review on Chitin and Chitosan Polymers: Structure, Chemistry, Solubility, Derivatives, and Applications. ChemBioEng Rev..

[18] Clark J., Clarke H.M., Cutler R., Cooper D., Edwards D.E., Rennie R., Ward D.E., Daintith J. (2008). A Dictionary of Chemistry.

[19] Krajewska B. (2004). Application of chitin- and chitosan-based materials for enzyme immobilizations: A review. Enzym. Microb. Technol..

[20] Feng F., Liu Y., Hu K. (2004). Influence of alkali-freezing treatment on the solid state structure of chitin. Carbohydr. Res..

[21] Jang M.K., Kong B.G., Jeong Y.I., Lee C.H., Nah J.W. (2004). Physicochemical characterization of α-chitin, β-chitin, and γ-chitin separated from natural resources. J. Polym. Sci. Part A Polym. Chem..

[22] Dutta P.K., Dutta J., Tripathi V.S. (2004). Chitin and chitosan: Chemistry, properties and applications. J. Sci. Ind. Res..

[23] Jain T., Kumar S., Dutta P.K. (2019). Carboxymethylchitin Nanocarrier (CMCNC): A Novel Therapeutic Formulation for Drug Release. Polym. Plast. Technol. Mater..

[24] Pokhrel S., Yadav P.N., Adhikari R. (2015). Applications of Chitin and Chitosan in Industry and Medical Science: A Review. Nepal J. Sci. Technol..

[25] Sridevi N.A., Karuppasamy K., Balakumar S., Shajan X.S. (2015). Mechanical and ionic conductivity studies on polymer electrolytes incorporated with chitin nanofiber for rechargeable magnesium ion batteries. Int. Journal Sci. Technol. Manag..

[26] Stephan A.M., Kumar T.P., Kulandainathan M.A., Lakshmi N.A. (2009). Chitin-Incorporated Poly(ethylene oxide)-Based Nanocomposite Electrolytes for Lithium Batteries. J. Phys. Chem. B.

[27] Pushpamalar V., Langford S.J., Ahmad M., Lim Y.Y. (2006). Optimization of reaction conditions for preparing carboxymethyl cellulose from sago waste. Carbohydr. Polym..

[28] Qi H., Liebert T., Meister F., Heinze T. (2009). Homogenous carboxymethylation of cellulose in the NaOH/urea aqueous solution. React. Funct. Polym..

[29] Ramos L.A., Frollini E., Heinze T. (2005). Carboxymethylation of cellulose in the new solvent dimethyl sulfoxide/tetrabutylammonium fluoride. Carbohydr. Polym..

[30] Spychaj T., Wilpiszewska K., Zdanowicz M. (2013). Medium and high substituted carboxymethyl starch: Synthesis, characterization and application. Starch-Stärke.

[31] Sun G.-Z., Chen X.-G., Li Y.-Y., Zheng B., Gong Z.-H., Sun J.-J., Chen H., Li J., Lin W.-X. (2008). Preparation of H-oleoyl-carboxymethyl-chitosan and the function as a coagulation agent for residual oil in aqueous system. Front. Mater. Sci. China.

[32] Tokura S., Nishimura S., Nishi N. (1983). Studies on chitin IX. specific binding of Calcium Ions by Carboxymethyl-Chitin. Polym. J..

[33] Ding F., Qian X., Zhang Q., Wu H., Liu Y., Xiao L., Deng H., Du Y., Shi X. (2015). Electrochemically induced reversible formation of carboxymethyl chitin hydrogel and tunable protein release. New J. Chem..

[34] Brugnerotto J., Lizardi J., Goycoolea F.M., Argüelles-Monal W., Desbrières J., Rinaudo M. (2001). An infrared investigation in relation with chitin and chitosan characterization. Polymer.

[35] Fung B.M., Khitrin A.K., Ermolaev K. (2000). An Improved Broadband Decoupling Sequence for Liquid Crystals and Solids. J. Magn. Reson..

[36] Shigenobu H., Kikuko H. (1991). Chemical Shift Standards in High-Resolution Solid-State NMR (1) 13C, 29Si, and 1H Nuclei. Bull. Chem. Soc. Jpn..

[37] Morcombe C.R., Zilm K.W. (2003). Chemical shift referencing in MAS solid state NMR. J. Magn. Reson..

[38] van Meerten S.G.J., Franssen W.M.J., Kentgens A.P.M. (2019). ssNake: A cross-platform open-source NMR data processing and fitting application. J. Magn. Reson..

[39] David G., Gontard N., Guerin D., Heux L., Lecomte J., Molina-Boisseau S., Angellier-Coussy H. (2019). Exploring the potential of gas-phase esterification to hydrophobize the surface of micrometric cellulose particles. Eur. Polym. J..

[40] Eyler R.W., Klug E.D., Diephuis F. (1947). Determination of Degree of Substitution of Sodium Carboxymethylcellulose. Anal. Chem..

[41] Tan S.C., Khor E., Tan T.K., Wong S.M. (1998). The degree of deacetylation of chitosan: Advocating the first derivative UV-spectrophotometry method of determination. Talanta.

[42] Cárdenas G., Cabrera G., Taboada E., Miranda S.P. (2004). Chitin characterization by SEM, FTIR, XRD, and13C cross polarization/mass angle spinning NMR. J. Appl. Polym. Sci..

[43] Sagheer F.A.A., Al-Sughayer M.A., Muslim S., Elsabee M.Z. (2009). Extraction and characterization of chitin and chitosan from marine sources in Arabian Gulf. Carbohydr. Polym..

[44] Straus S.K., Bremi T., Ernst R.R. (1997). Side-chain conformation and dynamics in a solid peptide: CP-MAS NMR study of valine rotamers and methyl-group relaxation in fully 13C-labelled antamanide. J.Biomol. NMR.

[45] Mobarak N.N., Ramli N., Ahmad A., Abdullah M.P. (2013). Development of Carboxymethyl Chitosan Based Green Polymer Electrolyte. J. Biobased Mater. Bioenergy.

[46] Ma G., Yang D., Zhou Y., Xiao M., Kennedy J.F., Nie J. (2008). Preparation and characterization of water-soluble N-alkylated chitosan. Carbohydr. Polym..

[47] Kittur F.S., Prashanth K.V.H., Sankar K.U., Tharanathan R.N. (2002). Characterization of chitin, chitosan and their carboxymethyl derivatives by differential scanning calorimetry. Carbohydr. Polym..

[48] Zong Z., Kimura Y., Takahashi M., Yamane H. (2000). Characterization of chemical and solid state structures of acylated chitosans. Polymer.

[49] Wan Y., Creber K.A.M., Peppley B., Bui V.T. (2003). Ionic conductivity of chitosan membranes. Polymer.

[50] Shamsudin I.J., Ahmad A., Hassan N.H., Kaddami H. (2015). Bifunctional ionic liquid in conductive biopolymer based on chitosan for electrochemical devices application. Solid State Ion..

[51] Shamsudin I.J. (2016). Investigation on the Effects of Imidazolium Ionic Liquids on the Derivatives of Chitosan and Kappa Carrageenan Based Biopolymer Electrolytes for Application in Electrochemical Devices. Faculty of Science and Technology.

